# Characterisation and localisation of the endocannabinoid system components in the adult human testis

**DOI:** 10.1038/s41598-019-49177-y

**Published:** 2019-09-19

**Authors:** John E. Nielsen, Antoine D. Rolland, Ewa Rajpert-De Meyts, Christian Janfelt, Anne Jørgensen, Sofia B. Winge, David M. Kristensen, Anders Juul, Frédéric Chalmel, Bernard Jégou, Niels E. Skakkebaek

**Affiliations:** 1 Department of Growth & Reproduction, and EDMaRC, Rigshospitalet, University of Copenhagen, Copenhagen, Denmark; 2 Univ Rennes, Inserm, EHESP, Irset (Institut de recherche en santé, environnement et travail) - UMR_S 1085, F-35000 Rennes, France; 3 Department of Pharmacy, Faculty of Health and Medical Sciences, University of Copenhagen, Copenhagen, Denmark; 4 Department of Neurology, Danish Headache Center, Rigshospitalet, University of Copenhagen, Copenhagen, Denmark

**Keywords:** Hormone receptors, Gonadal disorders

## Abstract

Heavy use of cannabis (marijuana) has been associated with decreased semen quality, which may reflect disruption of the endocannabinoid system (ECS) in the male reproductive tract by exogenous cannabinoids. Components of ECS have been previously described in human spermatozoa and in the rodent testis but there is little information on the ECS expression within the human testis. In this study we characterised the main components of the ECS by immunohistochemistry (IHC) on archived testis tissue samples from 15 patients, and by *in silico* analysis of existing transcriptome datasets from testicular cell populations. The presence of 2-arachidonoylglycerol (2-AG) in the human testis was confirmed by matrix-assisted laser desorption ionization imaging analysis. Endocannabinoid-synthesising enzymes; diacylglycerol lipase (DAGL) and N-acyl-phosphatidylethanolamine-specific phospholipase D (NAPE-PLD), were detected in germ cells and somatic cells, respectively. The cannabinoid receptors, CNR1 and CNR2 were detected at a low level in post-meiotic germ cells and Leydig- and peritubular cells. Different transcripts encoding distinct receptor isoforms (CB1, CB1A, CB1B and CB2A) were also differentially distributed, mainly in germ cells. The cannabinoid-metabolising enzymes were abundantly present; the α/β-hydrolase domain-containing protein 2 (ABHD2) in all germ cell types, except early spermatocytes, the monoacylglycerol lipase (MGLL) in Sertoli cells, and the fatty acid amide hydrolase (FAAH) in late spermatocytes and post-meiotic germ cells. Our findings are consistent with a direct involvement of the ECS in regulation of human testicular physiology, including spermatogenesis and Leydig cell function. The study provides new evidence supporting observations that recreational cannabis can have possible deleterious effects on human testicular function.

## Introduction

Possible adverse effects of cannabis (marijuana), a commonly used recreational drug, on male reproductive function, are a matter of concern. Frequent cannabis smoking has been repeatedly associated with decreased semen quality^1–4^ and increased risk of testicular germ cell cancer^5,6^. However, the mechanisms have not yet been elucidated. It is not known whether cannabis acts directly on the human testis or indirectly through disruption of the regulation of the gonadal function by the central nervous system (CNS). The main psychoactive component of cannabis, Δ9-tetrahydrocannabinol (THC), is an agonist of the endocannabinoid system (ECS), a well-conserved and ubiquitous signalling system, with multiple physiological roles. Among the natural agonists, anandamide (AEA) and 2-arachidonoylglycerol (2-AG) have been best characterised, particularly in CNS^7,8^. AEA is synthesised from phospholipid precursors by N-acyl-phosphatidylethanolamine-specific phospholipase D (NAPE-PLD) and 2-AG is synthesised from diacylglycerol (DAG) by a DAG-lipase (DAGL). The signalling of cannabinoids is mediated by several receptors, in particular two canonical receptors CNR1 and CNR2 (also known as CB1 and CB2, respectively), both structurally defined as G-protein-coupled receptors^7–9^. Possible alternative receptors have also been identified^10^. The ECS signalling is further regulated by a complex system of degrading enzymes; AEA is metabolised by the fatty acid amide hydrolase (FAAH) and 2-AG predominantly by the monoacylglycerol lipase (MGLL, also known as MAGL), but alternative metabolising pathways e.g. by oxidation have been described^7,8^.

The ECS has been best described in the CNS and in the immune system, but ECS is also involved in bone metabolism and reproductive functions^4,8,11^. Studies in rodents demonstrated the presence of CNR1 protein in germ cells, Leydig cells and possibly also Sertoli cells^11–15^. Other ECS components were identified in rodent testes mainly at the transcriptional level, including CNR2 and FAAH in Sertoli cells, and FAAH, NAPE-PLD, MGLL and DAGL in germ cells (reviewed in 11). Ablation of *Cnr1* in mice had systemic and local effects on reproductive function; including decreased serum LH and testosterone levels^16^, but also disturbance of chromatin remodelling in spermatids^13,14^.

Previous human investigations have focused on identification of ECS components in spermatozoa and association of their presence with selected parameters of sperm quality and function^1,2,17–22^. The ECS receptors were found in human spermatozoa, however with some discrepancies; while most studies demonstrated the presence of CNR1 alone^17,18^, some studies detected also in sperm the other canonical receptor, CNR2, albeit at a much lower expression level^18,19^. More recently, a distinct role of the ECS in sperm activation was documented by data showing that progesterone-induced degradation of 2-AG in the sperm plasma membrane by ABHD2 is necessary to activate CatSper channel^21^. It was also experimentally demonstrated that excess of 2-AG indeed impaired function of human spermatozoa *in vitro*, causing premature acrosome reaction and loss of sperm motility^22^.

In contrast to the numerous studies on human spermatozoa, very few studies of ECS have been carried out in human testis tissue. The existing data concern mainly the expression of transcripts of the ECS receptors and their variants, following the initial discovery of the cannabinoid receptor mRNA in the testis at the time of the human receptor’s cloning^23^. Alternative splice variants of CNR1, encoding non-canonical CB1A and CB1B protein isoforms were detected at a low expression levels in human testis tissue^24^. An additional transcript isoform of CNR2 (designed as CB2A, which encodes the same protein as the initially-characterized CB2B isoform), was also found to be expressed at a much higher level in human testis than in any other tissue, but this finding differed from the variant distribution in rodents^25^. Cellular localisation of these transcripts was not reported.

The aim of this study was to close some of the above-mentioned gaps of knowledge, and to investigate the expression and cellular localisation of the main ECS components, including the canonical receptors, their variants, and metabolic enzymes in adult human testes at the protein and transcriptional level. We were curious whether the ECS system found in spermatozoa is also present in earlier stages of spermatogenesis and whether human somatic cells contain some elements of the ECS. In this reference-providing study, we used immunohistochemistry (IHC) on human testis samples from tissue archives, and *in silico* analysis of the ECS transcripts and IHC data in existing datasets. For validation of the antibodies, we examined samples of human extra-testicular tissues, including epididymis. We also performed quantitative RT-PCR (qPCR) validation of the expression of ECS-receptors and their non-canonical isoforms. In addition, we investigated the presence of endocannabinoids AEA and 2-AG in testis tissue by matrix assisted laser desorption ionization (MALDI) imaging analysis. Taken together the data indicate that the ECS is present in the human testis and is likely to be directly involved in testicular function, particularly in regulation of spermatogenesis.

## Results

### Localisation of the ECS components in the adult testis at the protein level

**Figure 1:**
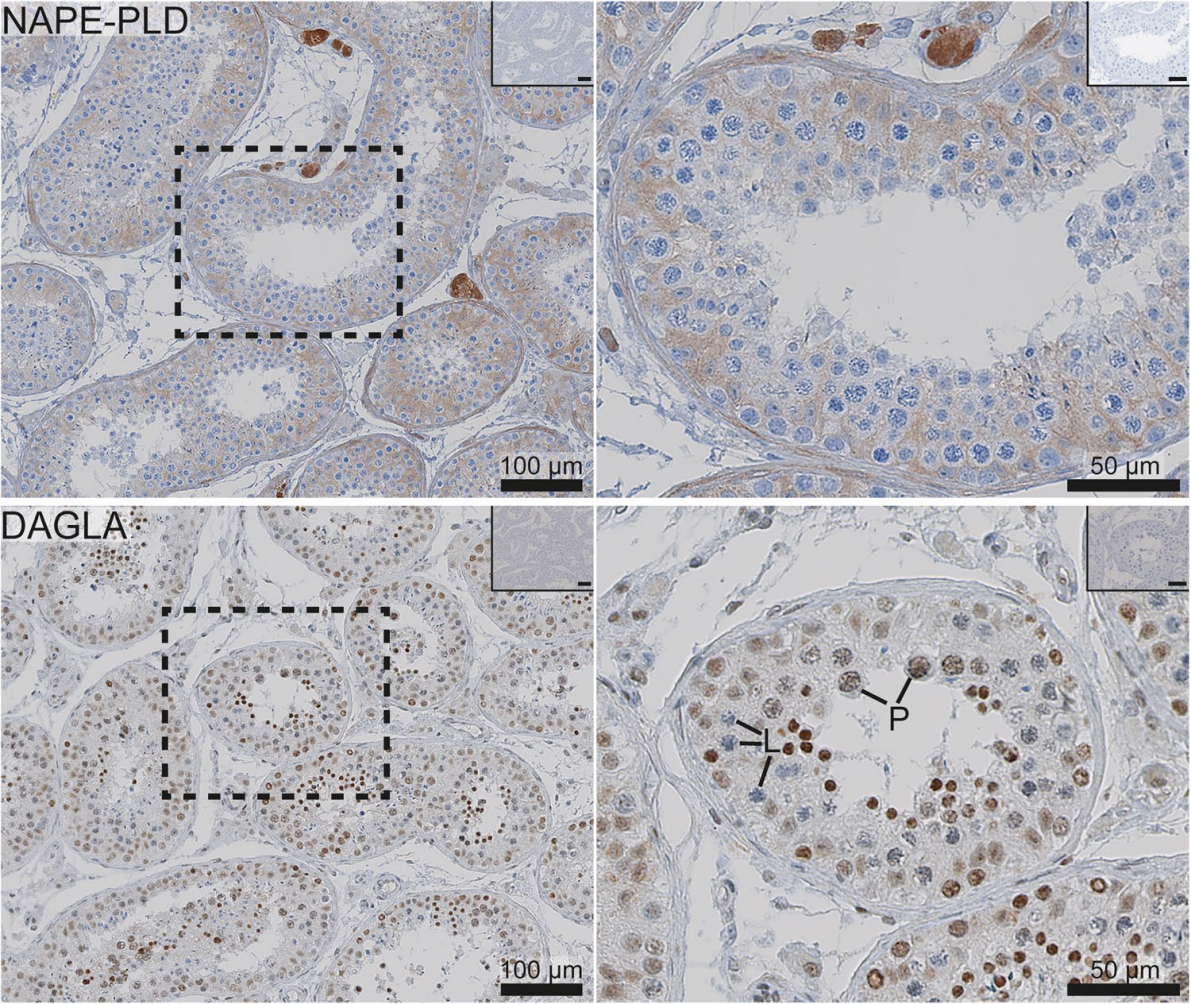
Immunohistochemical expression of the endocannabinoid-synthesising enzymes, NAPE-PLD and DAGLA, in the adult human testis. The images on the left show a general expression pattern, and a higher magnification of an area within a stippled line is shown on the right. All pictures show a low magnification image of negative controls in the right upper corner. NAPE-PLD was present in Leydig and Sertoli cells. DAGLA was present predominantly in the nuclei of germ cells, with only weak reaction detected in Sertoli and Leydig cells. L (leptotene) and P (pachytene)  indicate early and late spermatocytes, respectively.

**Figure 2:**
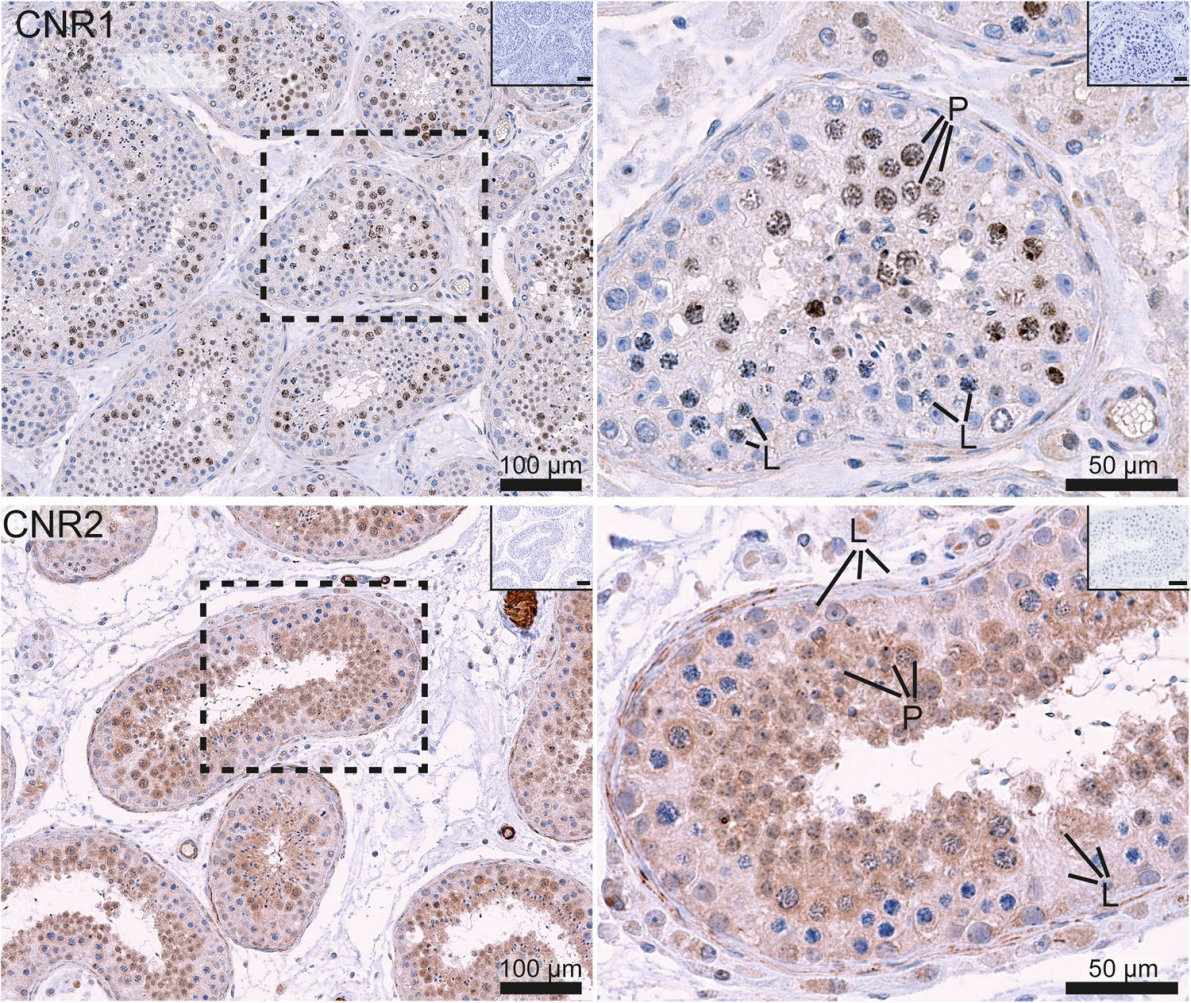
Immunohistochemical expression in human testis tissue of the two canonical EC receptors; CNR1 and CNR2. The images on the left show a general expression pattern, and a higher magnification of an area within a stippled line is shown on the right. All pictures show a low magnification image of negative controls in the right upper corner. CNR1 staining (upper images) is visible within nuclei of primary spermatocytes and in the cytoplasm of Leydig cells. CNR2 (bottom images) is localised in the cytoplasm of all types of germ cells, except early spermatocytes, and in somatic cells, especially in Leydig cells and peritubular cells. L (leptotene) and P (pachytene) indicate early and late spermatocytes, respectively.

**Figure 3:**
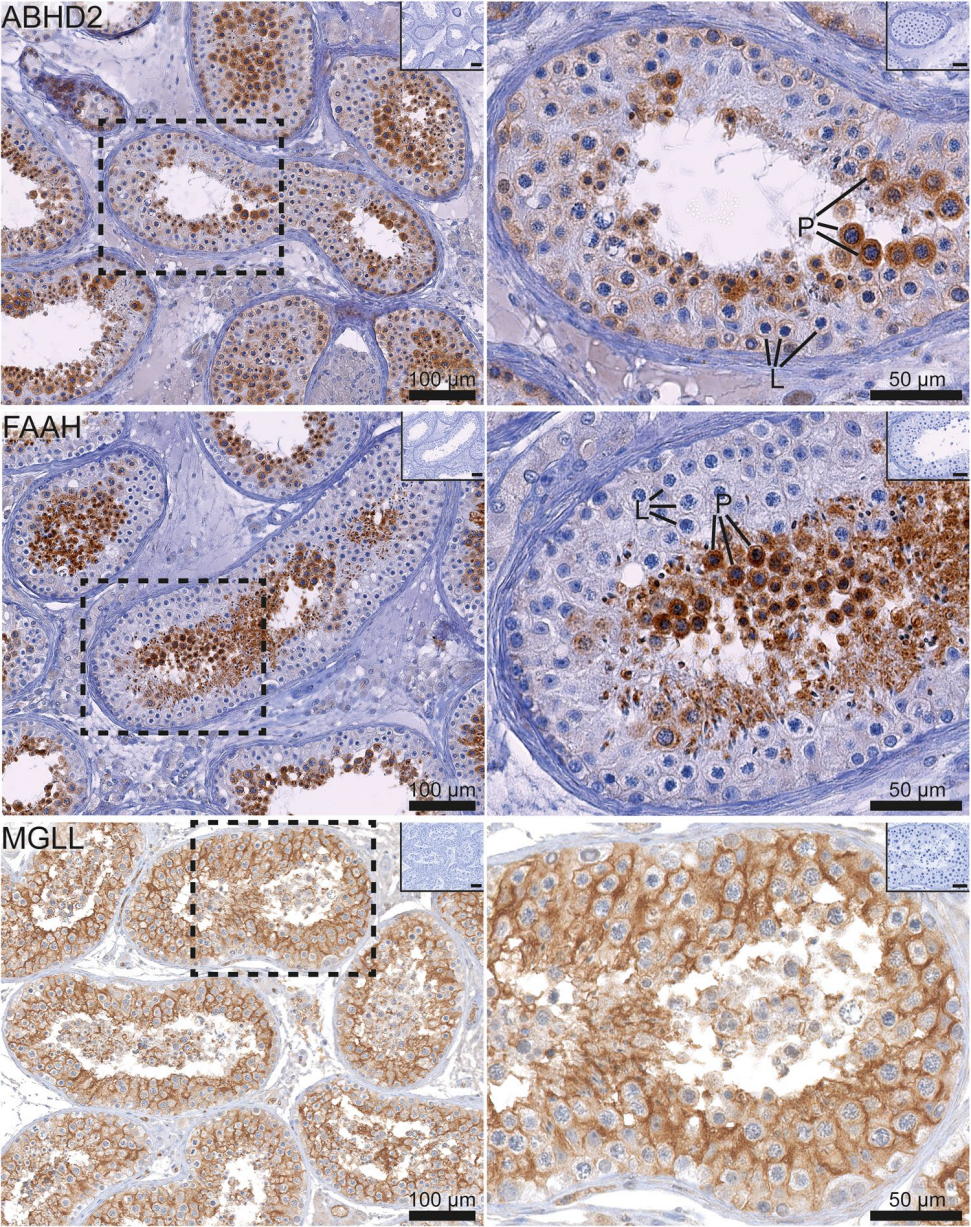
Immunohistochemical staining for the EC-degrading enzymes ABHD2, FAAH and MGLL in the human testis. The images on the left show a general expression pattern and the images on the right show a higher magnification of an area within a stippled line. All pictures show a low magnification image of negative controls in the right upper corner. Note that the ABHD2 (top images) is expressed in the cytoplasm of all stages of germ cell maturation, except early spermatocytes. FAAH reaction (middle row) is strongest in post-meiotic germ cells, including late spermatocytes and spermatids. MGLL protein (bottom images) is abundant in Sertoli cells. L (leptotene) and P (pachytene) indicate early and late spermatocytes, respectively.

We investigated by IHC the spatial organisation of the ECS components necessary for the ECS machinery to function. The IHC results in the testis are summarised in Table 1, separately for each cell type, and representative images are shown in Figs 1–3.

**Table 1:** Summary of the results showing predominant expression pattern of the ECS components in specific testicular cell types, grouped into germ cells and somatic cells. Immunohistochemical (IHC) data are shown in upper lines, with the subcellular protein localisation distinguished as nuclear (N) or cellular (C). Transcriptome data from RNA-sequencing (RNA-seq) for available cell types are listed underneath in *italics*. Note that the RNA-seq data for spermatocytes did not distinguish any specific stages, and that the expression of the receptor RNA isoforms was studied by qPCR.

ECS Component	N	Germ cells						Somatic cells			BV
		Spg	early-Spc	P-Spc	late-Spc	round-Spt	elongated-Spt	Sertoli	Peritub.	Leydig	
• Synthesising enzymes											
NAPE-PLD protein	14	−	−	−	−	−	−	C+ or +/−	C+/−	C+/++/−	C+
*NAPEPLD transcripts*		n.a.	+			++	n.a.	+	+	+	n.a.
DAGLA protein	11	N+/++/−	−	N+ (5/11)	N+ (5/11)	N+/++ (5/11)	−	N+ or +/−	−	N+/−	N +/−
*DAGLA transcripts*		n.a.	+			−	n.a.	+	+	+	n.a.
*DAGLB transcript*		n.a.	+			+	n.a.	+++	++	+	n.a.
• Receptors (canonical and isoforms)											
CNR1 protein	10	−	−	N++/+*	N++/+	N++/−	−	−	−	C+/−	−
*CB1 RNA isoform*		n.a.	n.a.	+		+	n.a.	−	−	+	n.a.
*CB1A RNA isoform*		n.a.	n.a.	+		+	n.a.	−	−	+	n.a.
*CB1B RNA isoform*		n.a.	n.a.	+		+	n.a.	−	−	−	n.a.
CNR2 protein	11	C+/−	−	C++	C++	C++	C++	−	C++	−	C+++
*CB2B RNA isoform*		n.a.	n.a.	−		−	n.a.	−	−	−	n.a.
*CB2A RNA isoform*		n.a.	n.a.	+		+	n.a.	−	−	−	n.a.
• Catabolic/degrading enzymes											
ABHD2 protein	13	C+/++/−	C+/−	C++	C++	C++	C++/+	−	−	C−/+	−
*ABHD2 transcripts*		n.a.	+++			++++	n.a.	++	++	+++	n.a.
FAAH protein	13	−	−	C++	C++	C++	C++	−	−	C−/+	−
*FAAH transcripts*		n.a.	++++			+++	n.a.	−	−	+	n.a.
MGLL protein	15	−	−	−	−	C/N−/+ (9/15)	C/N−/+ (9/15)	C+++6/+ (9/15)	−	C+/−	C++/−
*MGLL transcripts*		n.a.	+			+	n.a.	+++	++	++	n.a.

Abbreviations: Spg: spermatogonia, Spc: spermatocytes, P-Spc: pachytene spermatocytes, Spt: spermatids, Peritub: peritubular cells, BV: blood vessels. N: nuclear staining, C: Cytoplasmic staining, *localised intra-nuclear reaction. The IHC scoring is explained in the Methods section, ‘−‘ denotes no staining, ‘n.a.’ – cell type not available for transcriptional analysis. RNA-seq data are classified as strongly/moderately expressed (++++/+++/++), detectable (+) or undetectable (−).

The antibodies, listed in Table 2, were selected and validated as described in Materials & Methods and in Supplementary Information. Additional validation of all antibodies included testing in other tissues, including epididymis and efferent ducts (the tissues of importance for testis function) and several other tissue types, with known expression levels of ECS. These data are summarised in Supplementary Table S1, with representative examples shown in Supplementary Fig. S1.

**Table 2:** Antibodies used in the study, listed in alphabetical order.

Antibody name and targeted protein	Dilution	Pre-treatment buffer	Species	Provider (Catalogue Nr.)
ABHD2: Monoacylglycerol lipase (α/β-hydrolase domain-containing protein 2)	1:100	Citrate	Rabbit	Sigma (HPA005999)
CNR1 (CB1-R): Cannabinoid-binding receptor-1	1:400	Citrate	Rabbit	Abcam (ab23703)
CNR2 (CB2-R): Cannabinoid-binding receptor-2	1:400	TEG	Rabbit	Santa Cruz Biotechnology (sc-25494)
DAGL(A): Diacyglycerol lipase A	1:50	Citrate	Rabbit	Sigma (HPA062497)
FAAH: Fatty acid amide hydrolase	1:400	Citrate	Rabbit	Sigma (HPA007425)
MAGL (MGLL): Monoacylglycerol lipase	1:300	Citrate	Goat	Abcam (ab77398)
NAPE-PLD: N -acyl-phosphatidyl-ethanolamine-specific phospholipase D	1:100	TEG	Rabbit	Sigma (HPA024338)

Below, we briefly describe the protein expression pattern of the endocannabinoid-synthesising enzymes, the receptors and the endocannabinoid-metabolising enzymes. Because animal studies stipulated the involvement of ECS in distinct testicular functions, such as steroidogenesis, meiosis and spermiogenesis, we summarised the localisation of the ECS components in different compartments separately. The patterns of staining are first described in the seminiferous epithelium, with focus on different stages of germ cell maturation and in Sertoli cells, followed by the localisation of the ECS components in the interstitial compartment.

#### The seminiferous epithelium and maturation stages of germ cells

In germ cells, the localisation of several ECS components, was distinct in different maturation stages. The strongest expression was observed in post-meiotic germ cells. In spermatogonia a low-level cytoplasmic reaction of CNR2 was observed, and no CNR1 was detected (Fig. 2). CNR2 was more clearly expressed in the cytoplasm of late spermatocytes and round spermatids. CNR1 was also detected in germ cells, but in an unusual nuclear localisation. The strongest CNR1 expression was observed in pachytene spermatocytes (Fig. 2). An unusual nuclear reaction in germ cells (except early spermatocytes) was also noted in a subset of samples for DAGLA, a 2-AG-synthesizing enzyme, while no convincing staining for AEA synthesizing enzyme NAPE-PLD was detected in germ cells. The metabolising enzyme FAAH (2-AG-degrading) was abundantly expressed in the cytoplasm of late spermatocytes, round spermatids and late spermatids (Fig. 3). Another 2-AG-degrading enzyme, MGLL, was detected in nuclei of spermatids in a subset of samples, while in other samples the reaction was predominantly observed in Sertoli cells. ABHD2 (AEA-degrading) enzyme was expressed at a moderate level in spermatogonia, and more abundantly in late spermatocytes and spermatids (Fig. 3).

Sertoli cells, the somatic cells of the seminiferous epithelium, did not show convincing staining for the two canonical ECS receptors, and in general did not demonstrate a strong presence of the ECS components, except for NAPE-PLD and MGLL in a subset of samples. The AEA synthesizing enzyme, NAPE-PLD, showed a weak reaction in Sertoli cells (Fig. 1). The 2-AG-degrading MGLL displayed a heterogeneous reaction, with a strong staining in some samples, but rather weak in others, without any association with clinical diagnosis or quality of spermatogenesis. The MGLL reaction in Sertoli cells seemed to be weakest around spermatogonia. No other degrading enzymes, ABHD2 or FAAH, were detected in Sertoli cells.

#### Leydig cells and the interstitial compartment

The AEA-synthesising enzyme NAPE-PLD was strongly expressed in the cytoplasm of a subset of Leydig cells, while the 2AG-syntesizing DAGLA was not convincingly present, except a weak reaction in some cells (Fig. 1). The canonical ECS receptor, CNR1, showed a weak reaction, while CNR2 staining was detected quite clearly in peritubular cells and in a subset of Leydig cells. The peritubular cell reaction was though weaker than in epithelial and myoid cells of blood vessels, which are known to express CNR1 (Fig. 2). Virtually no expression of endocannabinoid degrading enzymes, ABHD2 and FAAH, was observed in the interstitial compartment, except some weak trace reaction in the cytoplasm of Leydig cells. MAGL/MGLL staining was weakly positive in Leydig cells and strongly positive in blood vessel walls (Fig. 3).

### Transcriptional profile of the ECS in isolated human testicular cell populations

To investigate the expression of the ECS components at the transcript level we first used existing RNA-sequencing (RNA-seq) data from human testicular cells^26^; (Supplementary Table S3). We found a consistent mRNA expression for both synthesising and degrading enzymes in distinct cell types, correlating to some extent with the protein data. 2-AG-synthesising *DAGLA* was detected at a lower level, in all cell types except spermatids, while *DAGLB* was highly expressed in Sertoli cells and peritubular cells. *NAPEPLD*, on the other hand, was mainly detected in spermatids. The expression of *FAAH* was strong and specific to spermatocytes and spermatids, supporting well the IHC data, while the expression of *MGLL* was very low in germ cells but clearly detectable in Sertoli- and Leydig cells. Additionally, *ABHD2* transcripts were readily detected in all cell types, with highest expression level in round spermatids.

**Figure 4:**
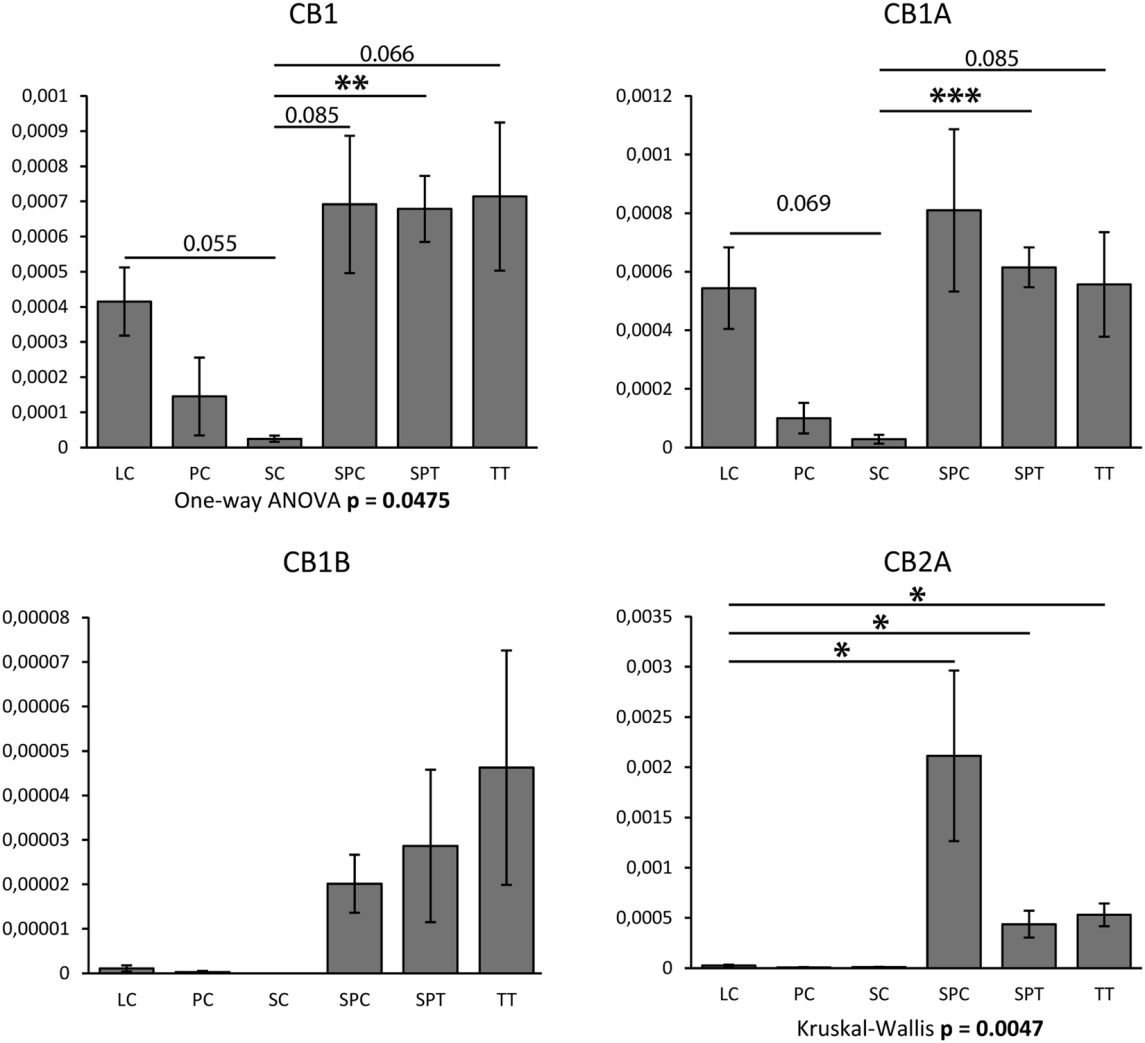
Expression profiles of transcript isoforms coding for CB1, CB1A, CB1B and CB2A. Quantitative PCR (qPCR) experiments were performed on cDNA from isolated human Leydig cells (n = 4; LC), peritubular cells (n = 2; PC), Sertoli cells (n = 2; SC), pachytene spermatocytes (n = 4; SPC), round spermatids (n = 4; SPT) and total testis tissue (n = 4; TT). Histograms represent mean expression (relative to *RPLP0*) ± SEM. Following evaluation of normality (Shapiro–Wilk test and D’Agostino’s K-squared test) and of homogeneity of variances (Brown-Forsythe test), global analysis of variances was achieved with One-way ANOVA (CB1 and CB1A) or with Kruskal–Wallis one-way analysis of variance (CB1B and CB2A). Pairwise comparisons were also performed using unpaired Student’s t-test (CB1 and CB1A) or with Mann–Whitney U test (CB1B and CB2A). One, two or three stars denote statistical significance with p < 0.05, p < 0,01 or p < 0.005, respectively. p-values close to significance are also indicated.

The expression levels of *CNR1* and *CNR2*, however, were below detection threshold in all cell types (maximum expression of 0.57 and 0.17 FPKM, respectively). We therefore undertook qPCR experiments (Fig. 4), for which we ensured to design primers specific for canonical isoforms as well as for non-canonical isoforms of both *CNR1*^24,25^ and *CNR2*^25^. A relatively low but consistent expression signal was found for both the canonical *CB1* isoform and the non-canonical *CB1A* isoform in Leydig cells, spermatocytes and spermatids. The non-canonical *CB1B* isoform was also detected, at very low levels in spermatocytes and spermatids, and at a slightly higher level in total testis samples, suggestive of a stronger expression in at least one additional testicular cell type. Regarding CNR2, the canonical *CB2B* isoform could not be detected at all. However, the non-canonical *CB2A* isoform was detected at low but consistent levels in spermatocytes, and to a lower extent in spermatids. Importantly, the purification and sequencing of PCR fragments confirmed the specificity of the expression signals for all four receptor isoforms. A summary of the data extracted from the RNA-seq human dataset is shown for each available cell type in Table 1, together with the protein data.

### Qualitative detection of endocannabinoid 2-AG in human testis tissue

**Figure 5:**
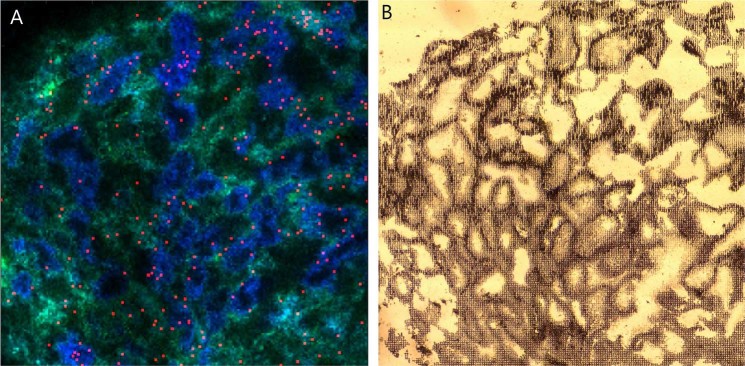
Detection of 2-AG in human testis. (**A**) MALDI imaging with blue colour showing a lipid associated with intra-tubular cells and green colour marking a lipid associated with interstitial cells, while red stain shows recognition of 2-AG dispersed among the tubules. (**B**) The frozen section after MALDI imaging showing the structure of the testis.

After detecting the presence of EC-synthesising enzymes, we then investigated whether the most common endocannabinoids, AEA and 2-AG are produced within the testis. By MALDI imaging methodology, we first analysed differentially distributed lipids in main testicular compartments. The PC (36:2) lipid (PC = phosphatidylcholine) detected as the sodium adduct at m/z 824.5566 was found to be associated with interstitial cells, while the PC (38:5) lipid detected as the sodium adduct at m/z 846.5409 was found within the intra-tubular compartment (Fig. 5). Using this basic separation of the two main compartments in the testis, we next made an examination for AEA and 2-AG. We were not able to find any detectable levels of AEA, likely due to the amount below the detection threshold. In contrast, we found 2-AG detectable as its potassium adduct at *m/z* 417.2402 – although at a very low signal intensity, with a localisation dispersed between the interstitial and tubular cells, as shown in Fig. 5. The extreme selectivity provided by the Orbitrap spectrometer could be observed by changing the imaged mass window to *m/z* 417.2422 (corresponding to an increase of the mass window by 0.002 Th), which made the distribution of 2-AG completely disappear. The signal was present in a very small mass window and disappeared upon such a small mass change, hence the signal was with very high probability due to 2-AG (or an isomer) and not an arbitrary background interference. The compound was also detected in adjacent tissue slices using the related desorption electrospray ionization mass spectrometric imaging (DESI-MSI) technique on a similar Orbitrap mass spectrometer with much better signal intensities, but at a resolution too low to spatially assign the origin of the signal.

## Discussion

Although several of the ECS components have been detected previously in human ejaculated spermatozoa^2,3,17–22^, our study is the first to demonstrate the presence of a specific pattern of this signalling pathway in various human testicular cell types, including germ cells at different stages of spermatogenesis, and in the somatic cells. We clearly demonstrated that the human testis has a machinery to synthesise and metabolise endocannabinoids, which are recognised by different testicular cell types (i.e. their receptors) within the seminiferous epithelium and the interstitial tissue. Interestingly, we detected a germ cell maturation stage–specific pattern of the ECS expression, with apparent paucity of most of the ECS components in early spermatocytes, distinct expression in pachytene spermatocytes, and the strongest expression in the post-meiotic spermatids.

The discovery of a CatSper channel activation in sperm by progesterone-dependent ABHD2 which removes 2-AG inhibiting this channel^21^, prompted us to investigate at which stage of germ cell maturation this enzyme and other elements of ECS appear, and whether 2-AG is produced in the testis tissue outside of sperm membranes. Surprisingly, we detected the presence of ABHD2 already in spermatogonia, but not in early spermatocytes, and this novel finding suggests an ECS function already in pre-meiotic germ cells, perhaps linked to commitment into meiosis, as suggested in rodent studies^27,28^. As expected, we found abundant ABHD2 protein and transcripts in spermatocytes and spermatids, and this is consistent with the subsequent function of this enzyme in transcriptionally inactive spermatozoa. We also found in germ cells an AEA-degrading enzyme, FAAH, with a robust expression at the transcriptional and protein level in late spermatocytes and spermatids, which supports previously reported findings of FAAH in ejaculated spermatozoa^2,18^. MGLL, which also metabolises 2-AG, was expressed in spermatids at much lower level, but was surprisingly abundant in Sertoli cells, however, with marked heterogeneity of expression between studied samples. We did not observe any association between MGLL expression level and the different stages of spermatogenesis, especially the presence of post-meiotic cells. We noticed though that the intensity of the MGLL staining was somewhat polarised, weakest near spermatogonia, suggesting that that pre-meiotic germ cells may need higher levels of 2-AG, as recently proposed in an elegant study of ECS components in rat testis^28^. Hence, we hypothesise that MGLL in Sertoli cells may be needed to metabolise 2-AG produced in post-meiotic germ cells and shed within residual bodies.

The proposed constant synthesis of 2-AG within sperm cell membranes to protect from premature sperm capacitation^21^ would also require presence of a diacylglycerol lipase, and its transcription during earlier stages of spermatogenesis. Indeed, the analysis of our RNA dataset revealed the presence *DAGLA* and *DAGLB* transcripts in both germ cells and somatic cells. The protein data, however, indicated the presence of DAGLA protein mainly in cell nuclei, and no suitable antibody was available for DAGLB. The public database, Human Protein Atlas^29^ reported the presence of DAGLA protein mainly in Leydig cells, hence more work is needed to firmly establish the cell type producing 2-AG within the testis.

In support of the presence of synthesizing enzymes in the testis, we detected their product, 2-AG in frozen testis tissue by MALDI-MS imaging. The low signal intensity in these experiments did not allow a firm conclusion concerning the cellular localisation. While such low intensities in other low-resolution mass spectrometry systems (e.g. ion traps and MALDI-TOF systems) might be dismissed as interferences with other background compounds, the risk of such interferences was greatly reduced by the use of a high-resolution Orbitrap mass spectrometer. With the same method we did not detect the other best-known endocannabinoid, AEA, reported by others to be present together with 2-AG in human seminal fluid^30–32^. We found though some supporting evidence for the synthesis of AEA in testicular tissue, as the required enzyme NAPE-PLD was detected in Leydig cells and in Sertoli cells. *NAPEPLD* transcripts were present in these somatic cell types but were also detected in round spermatids. One of the antibodies screened in the preliminary phase of the study detected also NAPE-PLD in round spermatids, but only in the nuclei. This enzyme was not studied previously in the human testis tissue, only in ejaculated spermatozoa, where NAPE-PLD protein was described in the post-acrosomal region and mid-piece^2,18^. Data available in public databases are also somewhat contradictory concerning this enzyme, so further studies concerning AEA synthesis in the testis are needed. By contrast, the existing data agree on the robust expression of the AEA-metabolising enzyme, FAAH, in late spermatocytes and in spermatids. This is consistent with a hypothesis that the level of AEA or another natural FAAH substrate must be kept very low during meiosis and post-meiotic maturation of germ cells.

It is possible that endocannabinoids, especially 2-AG, might act in transcriptionally inert spermatozoa in a non-genomic manner. In transcriptionally active cell types in the testis, the presence of several components of the ECS system suggests a genomic function, which is mediated by specific receptors. The existing information on the ECS receptors and their variants in the testis is fragmentary and sometimes contradictory, with apparent species differences^25^. CNR1 and CNR2, or CNR1 alone were described in murine Leydig cells and murine and frog germ cells, leading to a proposal that activation of CNR1 in Leydig cells is likely involved in steroidogenesis, while CNR2 in spermatogonia B might promote meiotic entry in mice^15,33,34^. In humans the data are scarce, and largely limited to expression at the RNA level in whole testis tissue extracts, including the testis-dominant expression of *CB2A* variant^24,25^. At the protein level previous human studies only investigated ejaculated spermatozoa; CNR1 alone was detected in one study^17^, while both CNR1 and CNR2 were found by other groups^19,20,34^.

We attempted to localise the EC receptors by IHC, but few antibodies are available, and none can distinguish the isoforms. We tested different antibodies (Supplementary Information), and three of the four CNR1 antibodies showed an unexpected localisation in germ cells within the nuclei. The most robust antibody showed an especially strong signal in dense structures in pachytene spermatocytes. Validation of the CNR1 antibody by Western blotting and by pre-absorption with corresponding blocking peptides confirmed its specificity (Supplementary Information). We were though unable to obtain enough isolated spermatocytes from patients to corroborate this finding in subcellular fractions, thus, we cannot completely exclude cross-reactivity.

While the presence of CNR1 in Leydig cells is consistent with previous studies in rodents, it is difficult to explain the presence of a G-protein coupled receptor (GPCR) within a cell nucleus, however there are some reports of GPCRs active in the nucleus^35^. Interestingly, an unexpected localisation of some translation initiation proteins but without actual translational activity, was detected in the XY body of murine spermatocytes^36^. The authors of that study proposed a “temporal poising” hypothesis that the XY body composed mainly of inert chromatin could be a storage place for factors needed to be active in round spermatids^36^. Such a way of a quick activation of the ECS during spermiogenesis is conceptually attractive but remains a hypothesis.

We can also speculate that CNR1 can have a discreet function within the cell nucleus of germ cells during transition from pre-meiotic spermatocytes to post-meiotic stages, including spermatids. Indeed, studies of *cnr1* knock-out mice evidenced problems with chromatin remodelling and histone displacement in spermatids, likely resulting from disturbed steroid hormone levels^13,14^. Interestingly, these mice also displayed premature motility of sperm in caput epididymis^37^, and a 2-AG gradient was noted along epididymis^38^. Studying ECS in sperm and epididymis was not the principal aim of our study. However, while testing the CNR1 antibody in other tissues we also found an interesting pattern in the epididymis and efferent ductules. A remarkably strong CNR1 reaction was observed in cell membranes of epithelium of the efferent ductules, while in epididymis groups of epithelial cells resembling clear cells were strongly and specifically stained, both in the cell membranes and the nuclei (see Supplementary Table S1 and Fig. S1). These distinct subcellular localisations of CNR1 in a subset of cells in testis, efferent ductules and epididymis need to be independently confirmed and the biological implications further investigated.

As far as the CNR2 is concerned, we found the protein moderately present in a subset of spermatogonia, but not in primary spermatocytes, and then re-appearing in post-meiotic germ cells. This is the first report of the presence of CNR2 receptor in human spermatogonia, and the pattern observed in our study is consistent with previous studies in mice, which demonstrated the involvement of the ECS in the mitosis-meiosis switch^15,34^. CNR2 was also present in the interstitial compartment, particularly in peritubular cells where the ECS signalling likely has another function.

A somehow contradictory result from our study is that protein data were not always supported by RNA data, including the virtual absence of gene expression for both CNR1 and CNR2 as evidenced by RNA-seq. We hypothesised that perhaps a low expression of poorly characterised truncated receptor isoforms could be an explanation of the observed atypical cellular localization of staining and the paucity of the canonical receptor transcripts. Indeed, we found by qPCR a low expression level of all three CNR1 transcript isoforms (CB1, CB1A and CB1B) and of one CNR2 transcript isoform (CB2A) in distinct testicular cells, mainly in spermatocytes and round spermatids, but had no data available for spermatogonia. Hence, we believe that a differential expression of the CNR1 variant truncated isoforms in human spermatocytes could be an explanation of the unusual localisation patterns. We could not confirm that on the protein level, because of lack of isoform-specific antibodies. Further studies at the level of receptivity of the ECS are therefore needed to clarify the respective role of each receptor isoform in the human adult testis.

**Figure 6:**
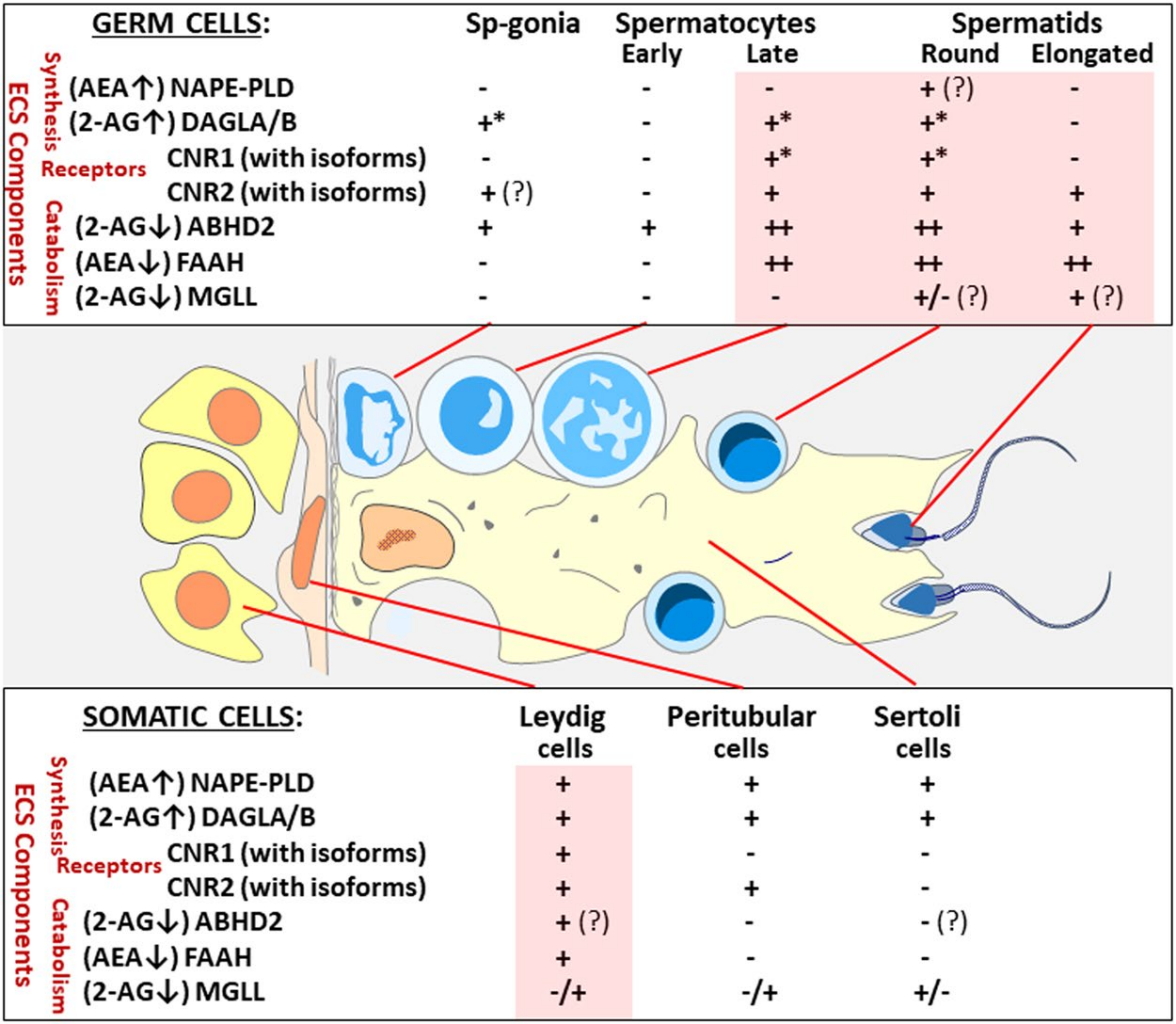
Graphical summary of the findings of this study illustrating the general expression patterns (transcripts and protein combined) of the main components of the endocannabinoid system (ECS) in different cell types of the human adult testis. All germ cell types are depicted with blue nuclei, and the somatic cell types with orange nuclei. The ECS components are grouped into the synthesising enzymes (with the main products shown in parentheses), the receptors and the catabolic enzymes (main substrates in parentheses). Germ cell maturation stages are shown in the upper panel, and the somatic cells at the bottom of the graph. Uncertain expression patterns, with some discrepancy between the transcript and protein levels, are denoted by question marks. The asterisks refer to uncertain subcellular location of the protein (nuclear). The testicular cell types with the greatest activity of the ECS are shaded in pink: the post-meiotic germ cells (upper panel) and Leydig cells (bottom).

In summary, we have characterised the localisation of main components of the ECS in the human testis and the emerging picture is illustrated schematically in Fig. 6. Within the seminiferous epithelium a balance between the synthesis and degradation of endocannabinoids is likely to be involved in the regulation of spermatogenesis. We noted that the transition from spermatogonia to early primary spermatocytes (i.e. from mitosis to meiosis) was associated with a relative silencing of the ECS receptors and metabolising enzymes. The opposite was observed in post-meiotic germ cells, with high expression levels of the ECS catabolising enzymes, likely needed to maintain a high turnover of endocannabinoids during spermiogenesis or provide their supply for use in spermatozoa outside the testis. The receptor signalling at these stages requires further investigations, but it nevertheless appears that CNR1 or its isoforms may be enriched in spermatocytes for an immediate action in specific functions related to chromatin changes during meiosis and spermiogenesis. Sertoli cells displayed lower levels of the ECS components, with absence of the receptors, perhaps related to the low proliferation of Sertoli cells in the adult testis. Peritubular cells also contain ECS, and express one of the canonical receptors, CNR2. The Leydig cells are characterised by a strong expression of the AEA-synthesising enzyme NAPE-PLD and the presence of both receptors, CNR1 and CNR2, in combination with a relative paucity of the degrading enzymes, possibly reflecting the participation of AEA in steroidogenesis.

In conclusion, the specific and differential expression patterns suggest a direct involvement of the ECS in the physiology of the human testis. The findings are relevant in relation to studies showing associations between a use of marijuana and changes in semen quality and reproductive hormone levels in young men^1–4,18–22^ as well as with regard to plans of cannabis legalisation^39^. Clearly, more studies of the endocannabinoid function in the human reproductive organs are warranted to address more comprehensively the health impact of cannabis.

## Materials and Methods

### Tissue samples

The testis tissue samples for the IHC analyses were excised from tumour-free periphery of orchiectomy specimens from 15 adult patients with testicular germ cell cancer. Only the specimens with tumours typical for young adults, i.e. derived from germ cell neoplasia *in situ* (GCNIS), either seminoma or nonseminoma, were included. None of the orchidectomy specimens contained sex-cord stromal cell tumours, e.g. Sertoli cell- or Leydig cell tumours. After pathologist’s evaluation, the remaining parts of the orchidectomy specimens were divided into small fragments (including tumour tissue, preserved testicular tissue, and epididymis), fixed overnight in GR fixative (modified Stieve fixative) or buffered formalin, and subsequently embedded in paraffin. The samples of preserved testicular parenchyma were evaluated by experienced pathologists to determine whether preinvasive GCNIS or any microinvasive tumour were present and to characterise spermatogenesis within the samples. Care was taken to select for this study tissue fragments with complete spermatogenesis including a good number of late spermatids, and without any pathological changes. The presence of GCNIS of micro-invasive tumour cells was excluded by IHC staining for specific markers^40^. Together with epididymis we also examined efferent ducts, which have not been previously reported in the literature with regard to the ECS expression. In addition, formalin-fixed and paraffin-embedded archival blocks of other human tissues from anonymous donors were included as controls. The following tissues with reported expression levels of ECS components were included: adrenals, skin, prostate and samples of placenta collected at term. For MALDI imaging analysis, frozen tissue samples were obtained from multi-organ donors.

### Ethical approval

A regional medical research ethics committee of the Capital Region of Copenhagen approved the use of human tissues stored in the biobank at Department of Growth and Reproduction for gene expression studies (permit number H-16019636). All patients have given informed consent for donating the residual tissues for research. The protocol for MALDI investigations was approved by the local ethics committees in Rennes and informed consent was obtained from all donors or their next of kin (CCPPRB Rennes; authorization 05/39–566; Agence de la Biomédecine; authorization #PFS09-015). All laboratory procedures and data evaluations have been performed in accordance with the Helsinki II Declaration and strict confidentiality.

### Antibodies

The primary antibodies used in this study, their sources and dilutions, and pre-treatment conditions are shown in Table 2. The antibodies were selected based on the previous performance in IHC of other tissues reported in the literature. Most of the commercial antibodies underwent extensive validation of their specificity by the suppliers, including Western blotting, and in all Sigma antibodies also IHC testing in a range of tissues, available in the public database Human Protein Atlas (www.proteinatlas.org), which also reports the presence of transcripts^29^. Several different CNR1 antibodies were tested before selecting the most robust one (Abcam) for the final study (Supplementary Table S2). Three of the four tested CNR1 antibodies demonstrated unusual nuclear localisation of staining in the testis. The selected antibody was additionally validated by Western blotting with and without a blocking peptide, and pre-absorption step in the IHC protocol (Supplementary Figures S2 and S3). We also did additional testing of the specificity of the antibodies by comparing staining patterns in testis tissue fixed in different fixatives, and in other human tissues known to have active ECS, including epididymis (Supplementary Table S1 and Fig. S1). Together with the epididymis we also examined efferent ductules, in which the ECS components have not been studied previously. The antibodies giving a staining pattern consistent with the localisation and role of the ECS components in these tissues were considered specific.

### Western blotting

Protein was isolated from frozen testis tissue samples containing normal seminiferous tubules without presence of malignant germ cells. Protein concentration of samples was estimated with The Micro BCA^TM^ Protein Assay Kit (Thermo Scientific). Western blotting was performed as previously described^41^ with 10 µg protein loaded in each lane. For detection of chemiluminescence, SuperSignal West Femto Chemiluminescent Substrate was used according to manufacturer’s instructions (Fisher Scientific, Roskilde, Denmark). Primary antibodies against CNR1 (Abcam) and β-Actin (Santa Cruz, used as loading control) were diluted 1:200, while secondary antibodies were diluted 1:1000. Blocking with immunizing peptide, corresponding to C terminal amino acids 461–472 of CNR1, was conducted according to the manufacturer’s protocol (Abcam), with one adjustment: a 10-fold higher amount of blocking peptide to primary antibody was used (instead of 2-fold). The experiment was conducted twice, and a representative example is shown in Supplementary Fig. S2.

### Immunohistochemistry

The IHC staining was conducted according to a standard indirect peroxidase method, as previously described, with some minor modifications^42^. In brief, tissue sections were subjected to heat-induced antigen retrieval in a pressure cooker (medical decloaking chamber, Biocare, Concord, CA, USA), either in 0.01 M citrate buffer (pH 7.4) or in TEG buffer (10 mM Tris, 1 mM EDTA, 0.05% Tween 20, pH 8.5) at 110 °C for 10–30 minutes. Endogenous peroxidase was blocked with 1% (v/v) H_2_O_2_ in methanol for 30 min. Unspecific staining was blocked using 0.5% skimmed milk in Tris-buffered saline (TBS) for 30 min. Sections were incubated overnight at 4 °C or room temperature with the primary antibodies diluted in TBS in a humidified chamber and then incubated for 30 min with the species-appropriate ImmPRESS HRP (peroxidase) secondary antibody. Between all steps (except after the blockage of unspecific staining) the sections were washed in TBS. Visualization was performed using ImmPACT DAB peroxidase (HRP) substrate or acetyl carbazole (ACE) (Vector Laboratories). The sections were subsequently counterstained with Meyer’s haematoxylin, and mounted on glass slides.

IHC controls: For negative controls, serial sections were processed with the primary antibody replaced by the dilution buffer alone. Sections of other tissues known to express the studied protein were used as positive controls. The immunostaining of other (extra-testicular) human tissues was performed with a slightly different protocol as these tissues were formalin-fixed. Pressure cooking heating was reduced to 10 minutes and antibodies were applied overnight at room temperature. For the polyclonal CNR1 antibody with the unusual localisation pattern in the testis (Abcam, ab23703), the IHC staining was supplemented by a pre-absorption step with blocking peptide, used by the manufacturer for immunisation (Abcam ab50542). The antibody in 1:400 dilution was thoroughly mixed with a 10-fold excess of the CNR1 blocking peptide and then applied on tissue sections. The rest of the staining protocol was as described above. The IHC with the peptide competition was repeated in three independent experiments, and a representative example is shown in Supplementary Fig. S3.

IHC evaluation and scoring: Three investigators evaluated systematically all IHC slides. The sections were first examined manually on a Nikon Microphot-FXA microscope, then were scanned on a slide scanner, NanoZoomer 2.0 HT (Hamamatsu Photonics, Herrsching am Ammersee, Germany) and analysed using the software NDPview version 1.2.36 (Hamamatsu Photonics). The observed IHC staining pattern and intensity was classified according to a pre-defined arbitrary scoring system: +++, strong staining in all cells of a given type in the sample; ++, staining in nearly all cells of a given type; ++/+, strong staining prevalent, but some weakly stained cells also visible; ++/−, strong staining present but negative cells also present; +/++/−, heterogeneous pattern with a mixture of strongly positive, weakly stained, and negative cells; +/++, majority of cells weakly stained, but focal strong staining present; +, weak staining overall; +/−, weak staining in limited areas; −/+, weak staining in single cells; −, no staining.

### RNA-sequencing data processing and transcript quantification

Human RNA-seq data from testicular cells^26^ were pre-processed as previously described^43,44^. Briefly, paired-end reads were first mapped on the human genome (hg19 release) using TopHat (2.0.10)^45^. The RefSeq transcript annotation (GTF format)^46^ was downloaded from the UCSC genome browser (https://genome.ucsc.edu/; 6 February 2017) and the abundance of corresponding genes in each sample was quantified using the Cufflinks suite^45^ in fragments per kilobase of exon model per million reads mapped (FPKM). All data are available at the ReproGenomics Viewer^47^; (http://rgv.genouest.org/app/#/). Gene expression data for the ECS components are provided in Supplementary Table S3.

### Quantitative PCR analysis

cDNAs were synthesized from the same RNA samples as those used for RNA-seq^26^, using the iScript™ Reverse Transcription Supermix for RT-qPCR (Bio-Rad) according to manufacturer’s instructions. Each gene was assessed in each sample in technical duplicates on 2 ng of cDNA using iTaq™ Universal SYBR® Green Supermix (Bio-Rad) and a CFX384 Touch™ Real-Time PCR Detection System (Bio-Rad) with default program (95 °C for 3 min followed by 40 cycles of 95 °C for 10 s, 60 or 64 °C for 30 s) including a melting curve step (65 °C to 95 °C with a 0.5 °C increment and a hold time of 5 s before reading plate). Previously published primers of *GAPDH* and *RPLP0* were used for normalisation purposes^48^ and results were analysed with the Bio-Rad CFX Manager™ software. Resulting PCR products were purified with the NucleoSpin® Gel 317 and PCR Clean-up kit (Macherey-Nagel) prior to sequencing using forward and reverse qPCR primers (Table 3 and Supplementary Figs S4 and S5).

**Table 3:** Primers used for qPCR experiments to characterise isoforms of endocannabinoid receptors. An asterisk (*) indicates that the primer spans an isoform-specific exon-exon boundary. The two nucleotides involved in the isoform-specific exon junction are underlined.

Target	Primer sequence (5′ → 3′)	Amplicon size	Annealing temperature
*CNR1* (CB1)	F: TCAGTACGAAGACATCAAAGGTG	85 bp	60 °C
	R: CTTCCCCTAAAGGAAGTTAAAGG		
CB1A	F: AGACATCAAAGGAGAATGAGGAG*	113 bp	60 °C
	R: GAGGGACAGGACTGCAATG		
CB1B	F: ACTGACCTCCTGGGAAGTCC *	84 bp	64 °C
	R: AATGTTCACCTGGTCTGCTG		
CB2A	F: GATTATGCCAGCCAGATGC	77 bp	64 °C
	R: GCTCGGTGAGTGAGAGGTG		

### Statistical analysis

Normal distribution of the qPCR data was first assessed for each isoform with a Shapiro–Wilk test as well as with a D’Agostino’s K-squared test. When normality was met (CB1 and CB1A), homogeneity of variances was further verified with the Brown-Forsythe test, prior to evaluating statistical differences with parametric tests, i.e. one-way ANOVA for global analysis of variances and unpaired Student’s *t*-test for pairwise comparisons. When normality was not met (CB1B and CB2A), non-parametric Kruskal–Wallis and Mann–Whitney U tests were used for global analysis of variances and for pairwise comparisons, respectively.

### Detection of endocannabinoids in human testis tissue by MALDI imaging analysis

A freshly isolated human testis sample was frozen at −25 °C using water as the only adhesive. The sample was cut into sections 20 µm thick using a Leica CM3050S cryo-microtome (Leica Microsystems, Wetzlar, Germany). Subsequently, the sections were thaw-mounted on glass slides and stored at −80 °C. On the day of analysis, the sample slide was taken directly from the freezer to a vacuum desiccator for 10 min prior to matrix assisted laser desorption ionization (MALDI) imaging analysis. For the analysis, the samples were spray-coated with a 30 mg/mL solution of 2,5-dihydroxybenzoic acid (DHB) matrix in methanol/water (50:50) containing 1% trifluoroacetic acid (TFA). 300 µL matrix solution was pneumatically sprayed with a flow rate of 20 µL/min from a 13 cm distance while the sample was rotating at 150 RPM, thereby forming a homogenous layer of fine matrix crystals over a circular area of approx. 30 mm diameter. The samples were analysed on a Thermo QExactive Orbitrap mass spectrometer, equipped with the AP-SMALDI10 ion source (TransMIT, Giessen, Germany). The mass spectrometer was operated in the positive ion mode (scan range m/z 225–900) at mass resolving power 140,000 at m/z 200, and a mass accuracy better than 1 ppm was ensured by using a matrix background peak as lock-mass. The samples were imaged using a pixel size of 10 µm. Subsequent microscope analysis showed laser ablation craters of approx. 7 µm diameter, confirming that oversampling was not performed. The raw data were converted to imzML using an imzML converter^49^ (available from www.maldi-msi.org), and MSiReader 0.09 was used to generate images^50^. The images were created using a bin width of 5 ppm.

## Supplementary information

Nielsen et al. Supplementary Information

## Data Availability

The data obtained in the course of this study are available as tables and figures in Supplementary Information. All initial raw RNA-sequencing data are available at the ReproGenomics Viewer^47^ (http://rgv.genouest.org/app/#/). Additional immunohistochemical images are available upon a direct contact with the corresponding author.
