# Author Correction: Characterisation and localisation of the endocannabinoid system components in the adult human testis

**DOI:** 10.1038/s41598-020-58153-w

**Published:** 2020-01-22

**Authors:** John E. Nielsen, Antoine D. Rolland, Ewa Rajpert-De Meyts, Christian Janfelt, Anne Jørgensen, Sofia B. Winge, David M. Kristensen, Anders Juul, Frédéric Chalmel, Bernard Jégou, Niels E. Skakkebaek

**Affiliations:** 1 Department of Growth & Reproduction, and EDMaRC, Rigshospitalet, University of Copenhagen, Copenhagen, Denmark; 2 Univ Rennes, Inserm, EHESP, Irset (Institut de recherche en santé, environnement et travail) - UMR_S 1085, F-35000 Rennes, France; 3 Department of Pharmacy, Faculty of Health and Medical Sciences, University of Copenhagen, Copenhagen, Denmark; 4 Department of Neurology, Danish Headache Center, Rigshospitalet, University of Copenhagen, Copenhagen, Denmark

Correction to: *Scientific Reports* 10.1038/s41598-019-49177-y, published online 19 September 2019

This Article contains errors. Reference 51 was inadvertently omitted and is given below as Reference 1.

1. Sun, X. *et al*. Genetic loss of Faah compromises male fertility in mice. *Biol Reprod*. **80**, 235–242 (2009).

As a result, in the Introduction,

“Studies in rodents demonstrated the presence of CNR1 protein in germ cells, Leydig cells and possibly also Sertoli cells^11,12,13,14,15^.”

should read:

“Studies in rodents demonstrated the presence of CNR1 protein in germ cells, Leydig cells and possibly also Sertoli cells^11,12,13,14,15^, CNR2 in germ cells and Sertoli cells, and FAAH mainly in spermatocytes and spermatids^1^”

“Ablation of *Cnr1* in mice had systemic and local effects on reproductive function; including decreased serum LH and testosterone levels^16^, but also disturbance of chromatin remodelling in spermatids^13,14^.”

should read:

“Ablation of *Cnr1* in mice had systemic and local effects on reproductive function; including decreased serum LH and testosterone levels^16^, but also disturbance of chromatin remodelling in spermatids^13,14^. Genetic ablation of *Faah* led to elevated levels of AEA and caused impairment of fertility, which was rescued in mice with simultaneous knock-out of *Cnr1*^1^.”

Additionally, within the Results,

“The peritubular cell reaction was though weaker than in epithelial and myoid cells of blood vessels, which are known to express CNR1 (Fig. 2).”

should read:

“The peritubular cell reaction was though weaker than in epithelial and myoid cells of blood vessels (Fig. 2).”

Furthermore, within the Discussion,

“CNR1 and CNR2, or CNR1 alone were described in murine Leydig cells and murine and frog germ cells, leading to a proposal that activation of CNR1 in Leydig cells is likely involved in steroidogenesis, while CNR2 in spermatogonia B might promote meiotic entry in mice^15,33,34^.”

should read:

“CNR1 and CNR2, or CNR1 alone were described in murine Leydig cells and murine and frog germ cells, leading to a proposal that activation of CNR1 in Leydig cells is likely involved in steroidogenesis, while CNR2 in spermatogonia B might promote meiotic entry in mice^15,33,34,1^.”

Finally, in Figure 2, the bottom right histology image is incorrectly labelled because of an inadvertent rotation of the image. The correct Figure 2 appears below as Figure 1.

**Figure 1:**
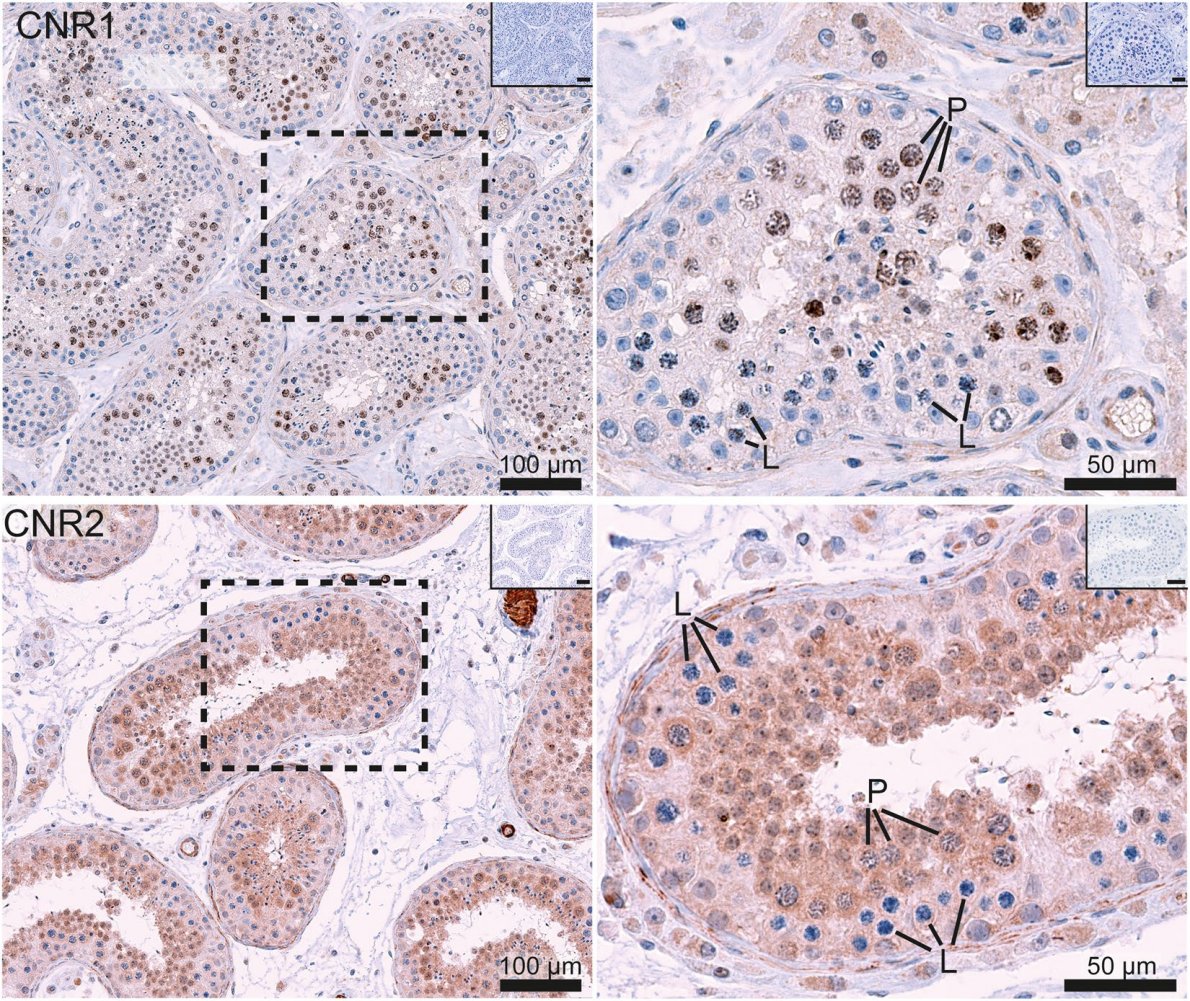
.

