# Supplementary material for: Characterisation and localisation of the endocannabinoid system components in the adult human testis
Source: Sci Rep. 2019 Sep 19;9:12866. doi: 10.1038/s41598-019-49177-y (PMC6753062; doi:10.1038/s41598-019-49177-y)

Supplementary Information

Characterisation and localisation of the endocannabinoid system components in the adult human testis

[1§]John E. Nielsen, [2§]Antoine D. Rolland, [1§]Ewa Rajpert-De Meyts, [3]Christian Janfelt, [1]Anne Jørgensen, [1]Sofia B. Winge, [2,4]David M. Kristensen, [1]Anders Juul, [2]Frédéric Chalmel, [2]Bernard Jégou and [1*]Niels E. Skakkebaek

[§]Equal contribution
*Corresponding author

1. Department of Growth & Reproduction, and EDMaRC, Rigshospitalet, University of Copenhagen, Denmark
2. Univ Rennes, Inserm, EHESP, Irset (Institut de recherche en santé, environnement et travail) - UMR_S1085, F-35000 Rennes, France
3. Department of Pharmacy, Faculty of Health and Medical Sciences, University of Copenhagen, Denmark
4. Department of Neurology, Danish Headache Center, Rigshospitalet, University of Copenhagen, Denmark

Content:

Supplementary Table S1: Antibody testing in epididymis and various extra-testicular human tissues.

Supplementary Figure S1: Examples of IHC patterns in human epididymis, efferent ductules and other tissues.

Supplementary Table S2: Additional CNR1 antibodies tested but not included in the final study.

Supplementary Figure S2A and S2B. Testing of the CNR1 antibody specificity by Western blotting.

Supplementary Figure S3. Pre-absorption control of immunohistochemical staining with the CNR1 antibody (Abcam) used in the final study.

Supplementary Table S3: RNA-sequencing data for ECS components in isolated populations of human adult testicular cells.

Supplementary Figure S4: Design of primers to assess for specific CNR1 isoforms by quantitative PCR.

Supplementary Figure S5: Design of primers to assess for specific CNR2 isoforms by quantitative PCR.

Supplementary Table S1: Testing of selected antibodies in various extra-testicular human tissues

The antibodies are listed in alphabetical order. The tissues were selected for testing according to the literature data suggesting either the presence or absence of a given ECS component. All extra-testicular tissues were fixed in buffered formalin and paraffin-embedded. Epididymis and efferent ducts, which are important for sperm export and maturation, were tested for all antibodies. Representative images are pictured in Figure S1. The immunohistochemical staining (IHC) was performed by a standard indirect immune-peroxidase protocol, with antigen unmasking by heating pre-treatment. Negative controls (with the primary antibody replaced by a dilution buffer) were negative in all cases. Subcellular localisation of the staining is marked as C: cytoplasm, M: cellular membrane, or N: nucleus. The IHC staining pattern was classified according to an arbitrary score: +++, strong staining in all cells; ++, staining in nearly all cells; ++/+, strong staining prevalent, but weakly stained cells also visible; ++/-, stained cells mixed with negative cells; +/++, majority of cells weakly stained, but focal strong staining present; +, weak staining overall; +/-, weak staining in some cells; -, no staining.

Antibody Source	Dilution (Buffer)	Tissue (n)	Reaction in cell types	Additional Comments
**ABHD2**<br>Sigma,<br>HPA005999	1:50 -<br>1:100<br>(Citrate)	Epididymis and efferent ducts (n=3)	<u>Epididymis (caput)</u>:<br>principal epithelial cells + C<br>clear cells +++ C/N<br><u>Efferent ducts</u>:<br>epithelial cells +/+- C	Smooth muscle cells and stromal cells negative in both components
		Adrenal gland (n=2)	zona glomerulosa +++ C<br>inner cortex + C<br>medulla: ++ C	
		Liver (n=1)	parenchyma +/++ C	
		Prostate (n=3)	glandular epithelium ++/+/- C<br>connective tissue +/- C	
**CNR1**<br>Abcam,<br>ab23703	1:50 -<br>1:200<br>(Citrate)	Epididymis and efferent ducts (n=3)	<u>Epididymis (caput)</u>:<br>principal/basal epithelial cells -/+ C<br>clear cells +++ C/N<br><u>Efferent ducts</u>:<br>epithelial cells +++/+/- C/M<br>cilia: +++	Staining much stronger in efferent ducts than in epididymis.<br>Smooth muscle and stromal cells negative in both components
		Adrenal (n=1) adult	zona glomerulosa ++/+ C<br>inner cortex +/++ C<br>medulla + C	
		Liver (n=1)	+/++ C	
		Prostate (n=1)	glandular epithelium ++/+ C, + N	
		Placenta (n=2)	syncythiotrophoblast ++/+ C	
		Seminal vesicle (n=1)	epithelium ++/+ C	
		Skin (n=1)	epidermis keratinocytes + N	
**CNR2**<br>Santa Cruz<br>Biotech.,<br>sc-25494	1:200 -<br>1:400<br>(TEG)	Epididymis and efferent ducts (n=3)	<u>Epididymis (caput)</u>:<br>principal epithelial cells ++/+/- C/M<br>stereocilia +++<br><u>Efferent ducts</u>:<br>epithelial cells ++/+- C	Blood vessels +++
		Adrenal gland (n=2)	zona glomerulosa +++ C<br>inner cortex + C<br>medulla +++ C	
		Prostate (n=2)	myoid cells ++ C<br>basal epithelium + C<br>connective tissue +++ C	

Table S1 - continued

**DAGLA**<br>Sigma,<br>HPA062497	1:50<br>(Citrate)	Prostate (n=2)	- (neg.)	
		Liver (n=1)	- (neg.)	
		Placenta (n=1)	syncythiotrophoblast ++/+ C/N	
		Skin (n=1)	epidermis ++/- N	Only positive in stratum granulosum
**FAAH**<br>Sigma,<br>HPA007425	1:100 -<br>1:400<br>(Citrate)	Epididymis and efferent ducts (n=3)	<u>Epididymis (caput)</u>:<br>principal/ basal epithelial cells ++/+ C/M<br>smooth muscle + C<br><u>Efferent ducts</u>:<br>epithelial cells ++/+ C/M<br>smooth muscle ++ C	Blood vessels +/- C
		Adrenal gland (n=1)	zona glomerulosa ++ C<br>inner cortex +/-<br>medulla ++ C	
		Prostate (n=3)	glandular epithelium ++/+ C	
		Seminal vesicle (n=1)	glandular epithelium +++ C<br>smooth muscle + C	
		Skin (n=1)	epidermis, hair follicles and sebaceous glands +++ C<br>hypodermis + C	
**MGLL (MAGL)**<br>Abcam,<br>ab77398	1:300<br>(Citrate)	Epididymis and efferent ducts (n=3)	<u>Epididymis (caput)</u>:<br>principal/basal epithelial cells –+ C<br>clear cells ++<br>stereocilia +<br><u>Efferent ducts</u>:<br>epithelial cells +/+- C/M<br>cilia +/++	Blood vessels +++ C
		Adrenal gland (n=2)	zona glomerulosa ++/+ C<br>inner cortex + C<br>medulla +/++ C	Blood vessels +++ C
		Prostate (n=3)	glandular epithelium +/- C<br>basal cells +++/++ C	Smooth muscle cells + C<br>Blood vessels +++ C.
		Placenta (n=1)	syncytiotrophoblast +/++/- C/N	++/+ C and N. Other cells from faint to negative
**NAPE-PLD**<br>Sigma,<br>HPA024338	1:50-<br>1:100<br>(TEG)	Epididymis and efferent ducts (n=3)	<u>Epididymis (caput)</u>:<br>principal epithelial cells +++ M<br><u>Efferent ducts</u>:<br>epithelial cells +/+- C (few K)	In epididymis the adluminal cell membrane of epithelial cells at the base of cilia strongly positive
		Seminal vesicle (n=1)	glandular epithelium ++/+/- C	

Supplementary Figure S1

Examples of IHC patterns in human epididymis, efferent ductules (eff. ducts) and other tissues. For the description of the antibodies and staining patterns, see Supplementary Table S1. The negative controls are shown as inserts in top right corners of all images. The three top rows (epididymis and efferent ducts) show immunohistochemical (IHC) staining developed using ACE (acetyl-carbazole, red) and the bottom row shows IHC staining developed with DAB (diaminobezidine, brown). The magnification of all images is the same; the bar corresponds to 50 µm.

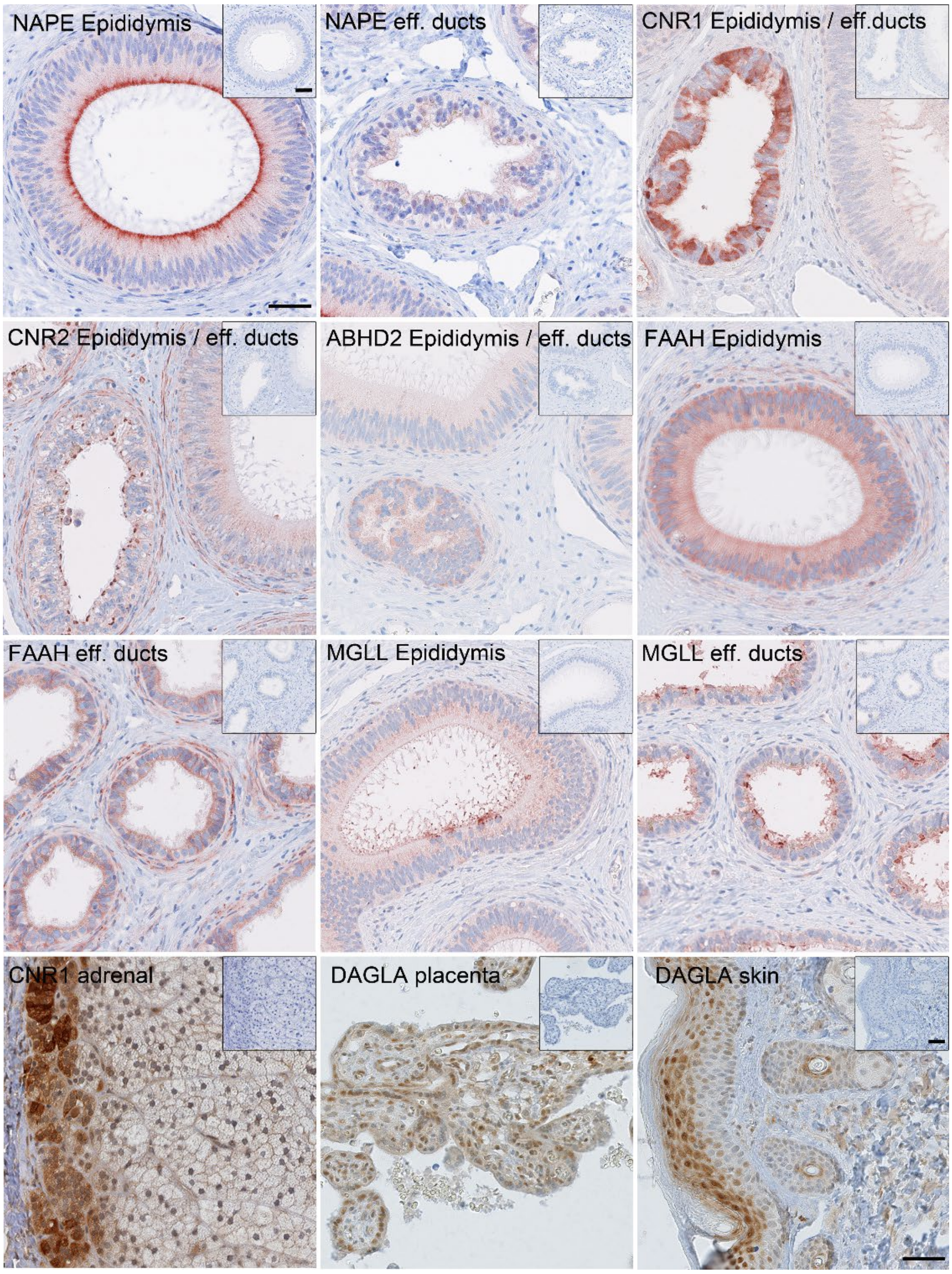

Supplementary Table S2: Anti-Cannabinoid Receptor 1 (CNR1) antibodies tested but not included in the final article.

Because of the unexpected nuclear localisation of CNR1 in spermatocytes and spermatids, additional antibodies against different epitopes of CNR1 protein were tested in the adult testis, epididymis and other tissues, fixed either in GR fixative or buffered formalin. The immunohistochemical staining (IHC) was performed essentially as described in the Material & Methods of the main article, but using several variations of the protocol, with different antibody dilutions and pre-treatment heating in different buffers (Citrate or TEG), in 1-3 different tissues of each type (testis N=3), and in 3-7 separate experiments. Controls with the primary antibody replaced by a dilution buffer were negative in all cases. The IHC gave to some degree similar results as the CNR1 antibody used in the main study (including nuclear localisation), but the quality of staining was substandard in our hands, often with difficult to interpret inconsistent reactions, hence, these antibodies were not included in the final study. The table below summarises the results of optimized experiments. Abbreviations: Spg: spermatogonia, Spc: spermatocytes, rSpt: round spermatids, eSpt: elongated spermatids Sertoli: Sertoli cells, Leydig: Leydig cells, PTC: peritubular cells. The subcellular localisation of the staining is marked as C: cytoplasm or N: nucleus. The IHC staining pattern was classified according to an arbitrary score as in Table S1 above.

**Antibody name and provider (Cat. Nr.)**	**Host Species**	**Epitope**	**Summary of the IHC staining patterns in the adult human testis, epididymis and other tissues**
CNR1<br>Abcam (Ab23703)	Rabbit	C-terminal amino-acids 461-472 of human CNR1	The antibody selected for the main study. The summary of the results in the adult human testis is presented in the main article and the pattern in other tissues is in Suppl. Table S1.
CNR1/CB1<br>LifeSpan BioSciences, Inc. (LS-B8253)	Rabbit	N-terminal amino-acids 73-122 of human CNR1	Testis: overall staining of all intratubular cell types, with Spg: +N, rSpt: +++C and perinuclear early acrosome-like body, eSpt: +/+++C and acrosome, Sertoli: ++C/N, Leydig: +/++/-)<br>Epididymis: epithelial cells: + (with scattered grains +++)<br>Efferent ducts: surface cilia: +++C, epithelial cells: ++/+C<br>Adrenals: wide-spread weak reaction in cortex: +C and medulla: +C<br>Skin: epidermis: +C, basal layer: +++N, peripheral: +N, hair follicles: +C /++/+N, sweat glands +1 N +/++/- (complex).
CNR1<br>Sigma-Aldrich/Merck (SAB4500345)	Rabbit	First intracellular loop, amino-acids 151-200 of human CNR1	Testis: overall staining of all intratubular cell types, with some Spg: +N/C, late Spc: +/++N/C, some rSpt: ++/+N, Sertoli: +C, Leydig: +C, PTC: ++/+C. The pattern inconsistent in different protocols.<br>Epididymis: epithelia: +C (cilia ++), myoid cells +/++/-C<br>Efferent ducts: +C (basal cells often -neg).<br>Adrenals: cortex: +C, medulla +/++C<br>Skin: epidermis +C, hair follicles +++/+C, sweat glands +C.
CNR1<br>Sigma-Aldrich (C2866)	Rabbit	Third cytoplasmic loop of human CNR1	Testis: wide-spread staining of all intratubular cell types, with strongest reaction in Sertoli: ++C, Spg: +C, Spc: + (perinuclear granules)/++N (a subset of Spc only), rSpt: + (perinuclear early acrosome-like body), Leydig: +C.<br>Epididymis: epithelial cells ++/+C<br>Efferent ducts: +++C<br>Adrenals: cortex: +/++ C, medulla: +/++/-C<br>Skin: epidermis -/+N, hair follicles +/++/-C, sweat glands single cells only ++C (complex pattern)

Supplementary Figure S2

A: Testing of specificity of the CNR1 antibody (Abcam, ab23703) by Western blotting with (right panel) and without (left) pre-absorbing the antibody with the corresponding blocking peptide (BP). Protein extracts from two specimens of normal adult testis (T1 and T2) were used. Predicted band size for the CNR1 antibody was 53 kDa and in both of the testis samples, two bands of approximately 55 and 45 kDa were detected. Addition of the blocking peptide resulted in disappearance of both observed bands, indicating that these contained the antigenic determinants. β-actin was included as a loading control.

B: Original and unprocessed full-length blots of the images shown in part A of the figure.

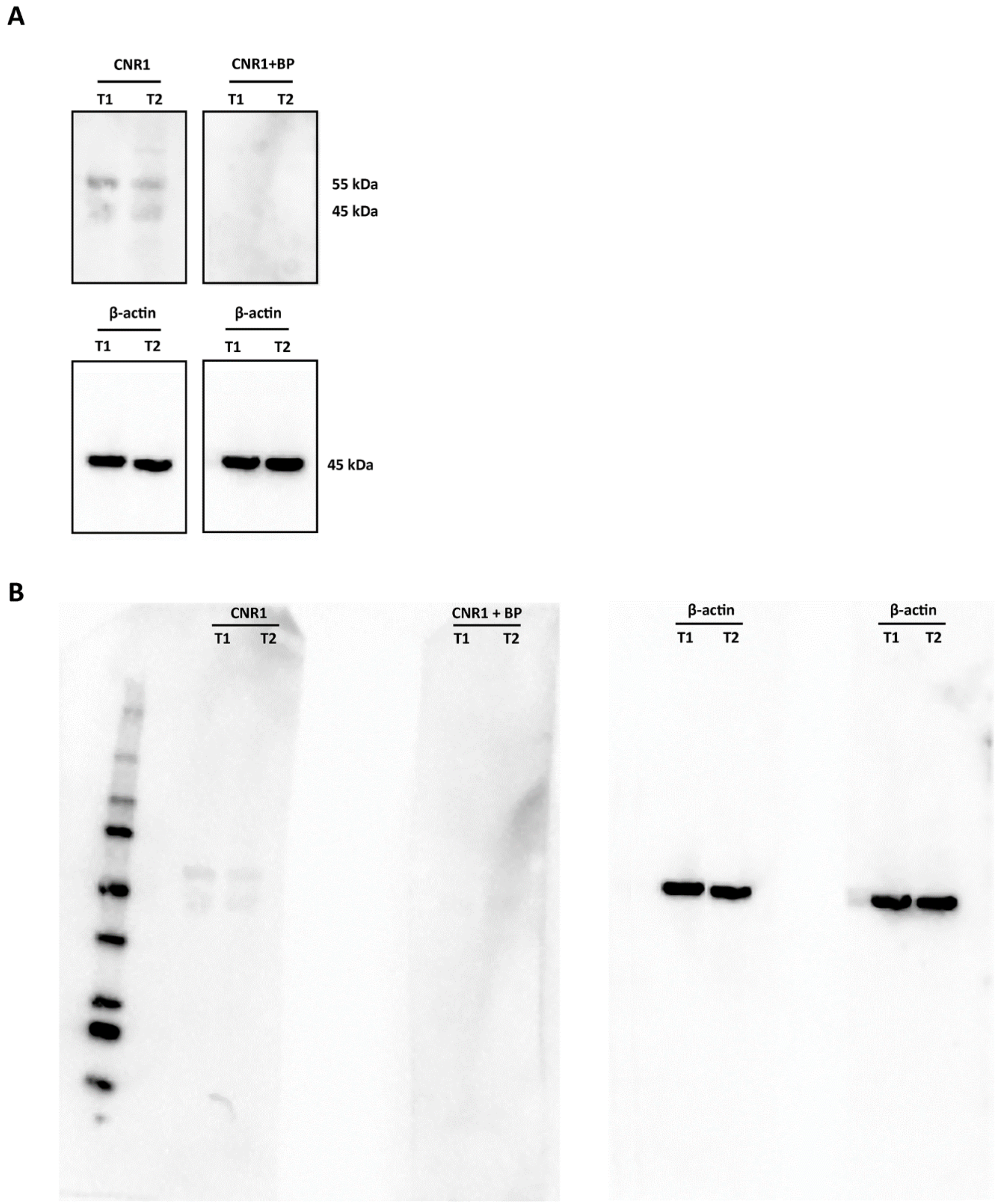

Supplementary Figure S3

Testing of the CNR1 antibody (Abcam, ab23703) by immunohistochemistry (IHC) in the human adult testis, with (right) and without (left) pre-absorption with an excess of the blocking peptide. A representative example of three independent IHC experiments is shown. A negative control without the primary antibody is shown in the insert on the bottom left.

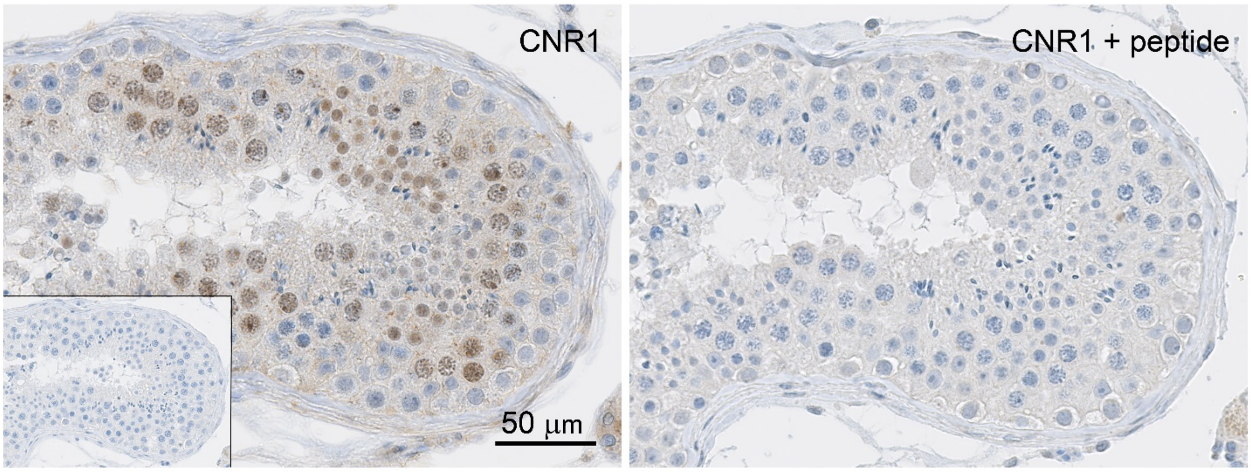

Supplementary Table S3: RNA-sequencing data for ECS components in human adult testicular cells.

Gene expression levels for ECS components as evidenced by RNA-seq in isolated human adult testicular cells (Jégou *et al.*, 2017). Expression levels for duplicate samples are averaged and provided as fragments per kilobase of exon model per million reads mapped (FPKM). LEY = Leydig cells ; PER = Peritubular cells ; SER = Sertoli cells ; SPC = Spermatocytes ; SPT = Spermatids ; TT = Total testes.

Gene Symbol	Entrez Gene ID	LEY	PER	SER	SPC	SPT	TT
ABHD2	**11057**	**36,151**	**21.196**	**12.022**	**27.191**	**50.651**	**13.348**
CNR1	**1268**	**0,178**	**0.004**	**0.007**	**0.199**	**0.131**	**0.566**
CNR2	**1269**	**0,052**	**0.039**	**0.000**	**0.209**	**0.062**	**0.158**
DAGLA	**747**	**2,068**	**1.193**	**1.212**	**1.365**	**0.412**	**1.459**
DAGLB	**221955**	**6,686**	**19.105**	**31.805**	**7.839**	**3.036**	**4.997**
FAAH	**2166**	**1,646**	**0.441**	**0.362**	**94.717**	**51.980**	**22.098**
NAPEPLD	**222236**	**3,676**	**2.098**	**1.841**	**4.131**	**11.903**	**9.036**
MGLL	**11343**	**47,032**	**29.819**	**66.187**	**0.719**	**0.765**	**3.851**

Reference:

Jégou, B., Sankararaman, S., Rolland, A.D., Reich, D., Chalmel, F. Meiotic genes are enriched in regions of reduced archaic ancestry. *Mol. Biol. Evol*. **34,** 1974-1980 (2017)

Supplementary Figure S4: Design of primers to assess for specific CNR1 isoforms by qPCR.

A. A snapshot from the UCSC genome browser at the human CNR1 locus (chr6:88,139,371-88,166,908) showing the NCBI Refseq gene annotation as well as the GENCODE comprehensive transcript set.

B-D. Three closer snapshots within the CNR1 locus (chr6:88,144,893-88,145,238) showing the amplicons (black bars) and the localization of forward (Fw.) and reverse (Rev.) primers (in red) for CB1 (B), CB1A (C) and CB1B (D). Red dashed lines show the isoform-specific introns spanned by the CB1A and CB1B forward primers. Note that for CB1 (B) the forward primer cannot hybridize to the CB1A isoform while the reverse primer cannot hybridize to the CB1B isoform. All three amplicons were confirmed by Sanger-sequencing using the forward primer as well as the reverse one.

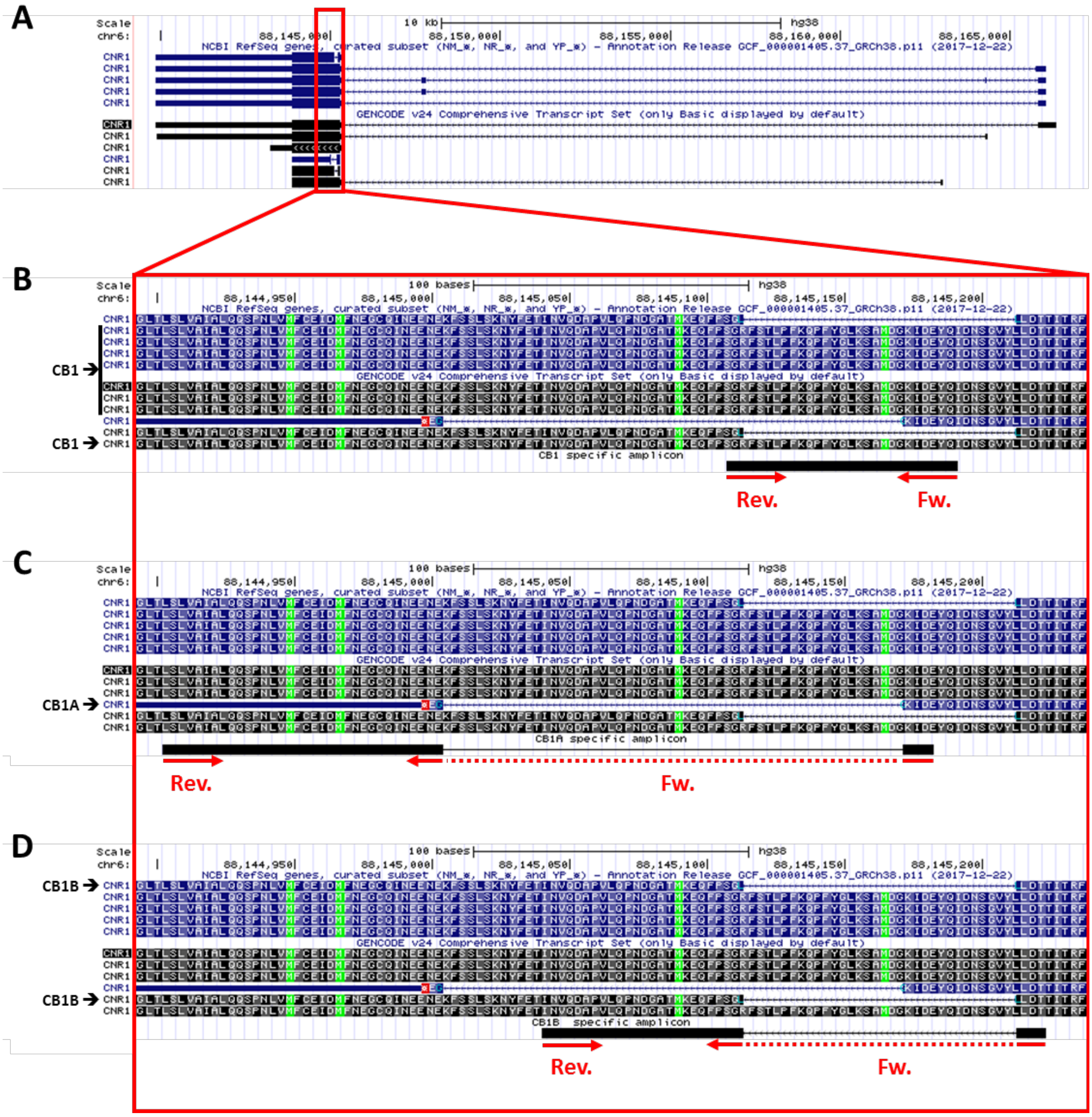

Supplementary Figure S5: Design of primers to assess for specific CNR2 isoforms by quantitative PCR.

A snapshot from the UCSC genome browser at the Human CNR2 locus (chr1:23,869,093-23,959,739) showing the NCBI Refseq gene annotation, the GENCODE comprehensive transcript set and the previously described testis-dominant CNR2 isoform encoding for CB2A (top-track; https://www.ncbi.nlm.nih.gov/nuccore/EU517121.1). The CB2-specific amplicon (red rectangle) is amplified thanks to primers flanking intron 1 of the canonical isoform, while the CB2A-specific amplicon (blue rectangle) is amplified thanks to primers flanking intron1 of the non-canonical isoform.

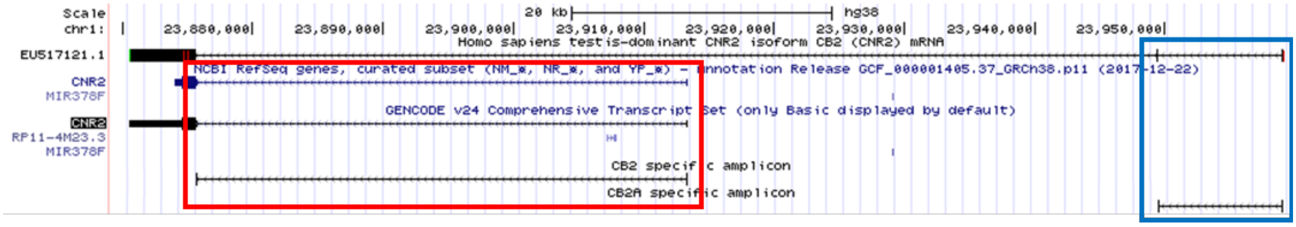

Supplement: Supplementary file 1 — Nielsen et al. Supplementary Information [file 41598_2019_49177_MOESM1_ESM.pdf]
